# Integrated Genomic and Proteomic Analyses of High-level Chloramphenicol Resistance in *Campylobacter jejuni*

**DOI:** 10.1038/s41598-017-17321-1

**Published:** 2017-12-05

**Authors:** Hui Li, Yingyu Wang, Qin Fu, Yang Wang, Xiaowei Li, Congming Wu, Zhangqi Shen, Qijing Zhang, Peibin Qin, Jianzhong Shen, Xi Xia

**Affiliations:** 1 Beijing Advanced Innovation Center for Food Nutrition and Human Health, College of Veterinary Medicine, China Agricultural University, Beijing, 100193 P.R. China; 2 Beijing Key Laboratory of Diagnostic and Traceability Technologies for Food Poisoning, Beijing Center for Disease Prevention and Control, Beijing, 100013 P.R. China; 3 Department of Veterinary Microbiology and Preventive Medicine, College of Veterinary Medicine, Iowa State University, Ames, IA 50011 USA; 4 Shanghai AB Sciex Analytical Instrument Trading Company Limited, Beijing, 100015 P.R. China

## Abstract

*Campylobacter jejuni* is a major zoonotic pathogen, and its resistance to antibiotics is of great concern for public health. However, few studies have investigated the global changes of the entire organism with respect to antibiotic resistance. Here, we provide mechanistic insights into high-level resistance to chloramphenicol in *C. jejuni*, using integrated genomic and proteomic analyses. We identified 27 single nucleotide polymorphisms (SNPs) as well as an efflux pump *cmeB* mutation that conferred modest resistance. We determined two radical S-adenosylmethionine (SAM) enzymes, one each from an SNP gene and a differentially expressed protein. Validation of major metabolic pathways demonstrated alterations in oxidative phosphorylation and ABC transporters, suggesting energy accumulation and increase in methionine import. Collectively, our data revealed a novel rRNA methylation mechanism by a radical SAM superfamily enzyme, indicating that two resistance mechanisms existed in *Campylobacter*. This work provided a systems biology perspective on understanding the antibiotic resistance mechanisms in bacteria.

## Introduction

*Campylobacter jejuni* is a major zoonotic pathogen and the leading bacterial cause of food-borne gastroenteritis worldwide^1,2^. Although most *C. jejuni* infections are clinically mild and self-limiting, the bacterium may cause severe infections in immunocompromised patients as well as serious post-infection complications such as Guillain-Barre syndrome and Miller-Fisher syndrome^3,4^. Therapeutic intervention is imperative in patients with severe or long-lasting infections, or with compromised immune systems^5^. However, the antimicrobial resistance of *C. jejuni* has increased significantly over the past decades and has become a major threat to public health^6,7^.

The use of antibiotics for growth promotion in livestock is of particular concern, because low doses of antibiotics when applied over long periods can create a breeding ground for the emergence of resistant bacteria. Apart from its use in both human and veterinary practice to treat infections, chloramphenicol (CAP) was once a widely used feed additive for food-producing animals, owing to its low cost and effectiveness against a wide variety of gram-positive and gram-negative bacteria. Nowadays, the use of CAP is limited to a small number of life-threatening infections in humans and is banned in many countries in animal husbandry because of its adverse effects^8,9^. Nevertheless, the detection rate of CAP-resistant *Campylobacter* is still high in some countries^10,11^. CAP binds directly to the 50 S ribosomal subunit and inhibits peptide bond formation by interacting with the peptidyl transferase center^12^. Previously reported bacterial resistance mechanisms to CAP included drug acetylation^13^, efflux pumps^14–17^, and methylation of rRNA by *cfr* ^18^.

In our previous work, a point mutation, G2073A, was identified in 23 S rRNA in CAP-selected mutants of *C. jejuni* ^19^. However, further study was needed to investigate whether this point mutation alone was sufficient to confer high-level resistance to CAP in the *C. jejuni* mutants. Here, we aimed to characterise the global changes in CAP-resistant mutants of *C. jejuni*, using integrated genomic sequencing and quantitative proteomic analysis. This work defined a previously unrecognized mechanism underlying high-level resistance to CAP in *C. jejuni*, demonstrating the usefulness of the systems biology approach in elucidating drug resistance mechanisms.

## Results

### Involvement of multiple mutations in CAP resistance

**Figure 1:**
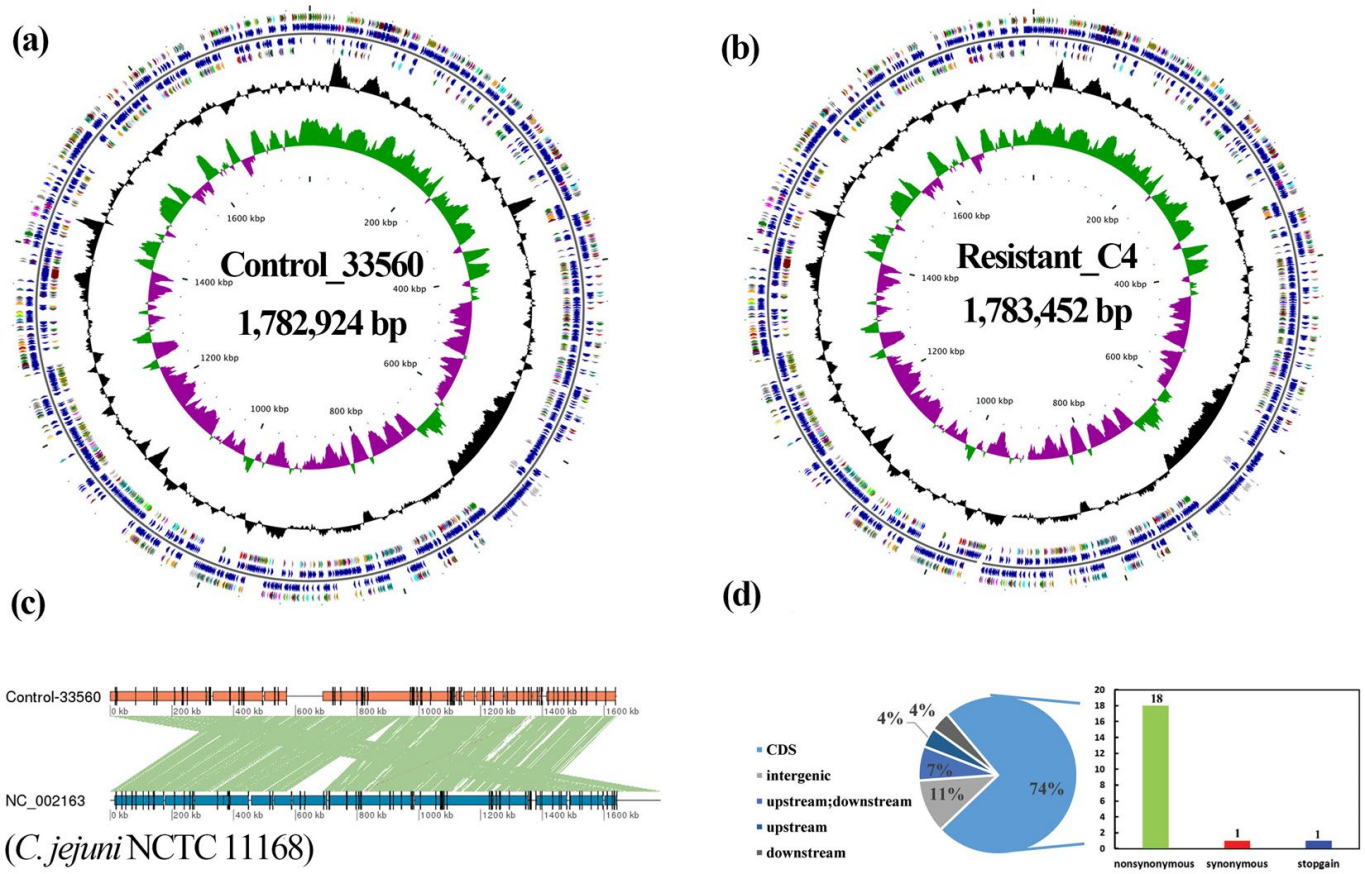
Complete genomes and comparative genomic analysis of parental strain and CAP-resistant strain. Circular representation of chromosome from *C. jejuni* ATCC 33560 (**a**) and CAP-resistant strain (**b**). Circles (from the center toward periphery) indicate the following: first, GC skew; second, G + C content; third, ORFs predicted on the minus strand; fourth, putative gene, rRNA and tRNA in the minus strand; fifth, putative gene, rRNA and tRNA in the sense strand; sixth, COG annotations of genes in the sense strand. (**c**) Co-linear analysis of genome between *C. jejuni* ATCC 33560 and *C. jejuni* NCTC 11168 using MUMmer. The green connecting bars indicated high sequence identity and the red bars a reverse orientation. (**d**) Number of mutant loci and categories of mutations in CAP-resistant strain.

The CAP-resistant C4 strain and the parental strain *C. jejuni* ATCC 33560 were completely sequenced (GenBank accession number, CP019838), and both strains contained a single contig of 1.78 Mbp with no plasmids (Fig. 1a,b). Genomic annotation of the parental strain indicated the presence of 1,869 putative coding sequences (Table S1). A co-linearity analysis revealed that *C. jejuni* ATCC 33560 had similar genome characteristics to those of *C. jejuni* NCTC 11168 (Fig. 1c). No known antibiotic resistance genes were identified in the resistant strain. Twenty-seven single nucleotide polymorphisms (SNPs) were identified between the mutant and parental strains (18 nonsynonymous, 1 stop-gain, 1 synonymous, and 7 noncoding; Fig. 1d and Table 1), among which *cje1821* exhibited three nonsynonymous substitutions. The two substitutions (G2073A and G74D) we found in our previous report were also identified here (Table 1)^19^. To investigate the role of these SNPs in the resistance mechanism, we constructed gene-knockout mutant strains of the 17 genes with nonsynonymous and stop-gain SNPs. Five genes (*cje0220*, *cje0275*, *cje*11*67*, *cje1432*, and *cje1821*) were successfully knocked out in resistant C4 strains. Then the resistance phenotype of these five knockout strains (Δcje0220, Δcje0275, Δcje1167, Δcje1432, and Δcje1821) was determined. Antibiotic susceptibility tests showed that the minimum inhibition concentrations (MICs) of CAP remained at 256 μg/mL for Δcje0220 and Δcje1167, whereas for Δcje0275, Δcje1432, and Δcje1821, the MIC was decreased to 64 μg/mL. These findings indicated that the high-level resistance to CAP in *C. jejuni* was mediated by multiple gene alterations.

**Table 1:** SNPs in CAP-resistant strain.

Reference	Alteration	Gene	Function	Putative protein/RNA	Mutation type	Amino acid change
G	A	*cje0035, cje0036*	intergenic			
T	C	*cje0036*	downstream			
G	A	*cje0075*	CDS	transcription termination/antitermination protein NusG	nonsynonymous	c.G433A:p.E145K
G	A	*cje0220*	CDS	NOL1/NOP2/sun family protein	nonsynonymous	c.G331A:p.G111S
G	A	*cje0275*	CDS	peptidyl-prolyl cis-trans isomerase	nonsynonymous	c.G1483A:p.G495R
G	A	*cje0289*	CDS	putative ribosomal pseudouridine synthase	nonsynonymous	c.G494A:p.S165N
G	A	*cje0332,cje0333*	intergenic			
T	C	*cje0333*	upstream			
A	G	*cje0473, cje0474, cje0475*	upstream, downstream			
G	T	*cje0766*	CDS	NAD(P)H-dependent oxidoreductase	stopgain	c.G160T:p.E54X
C	T	*cje0886*	CDS	30 S ribosomal protein S2	nonsynonymous	c.G118A:p.D40N
C	T	*cje1065*	CDS	hypothetical protein	synonymous	c.G423A:p.E141E
C	T	*cje1119*	CDS	putative cytochrome P450	nonsynonymous	c.G221A:p.S74N
C	T	*cje1167*	CDS	radical SAM protein	nonsynonymous	c.G113A:p.R38K
C	T	*cje1427*	CDS	50 S ribosomal protein L4	nonsynonymous	c.G221A:p.G74D
C	T	*cje1432*	CDS	16 S rRNA dimethyltransferase	nonsynonymous	c.G769A:p.D257N
G	A	*cje1486, cje1487*	intergenic	23 s rRNA	.	c.G2073A
G	A	*cje1496*	CDS	GTP-binding protein TypA	nonsynonymous	c.C1561T:p.L521F
A	-	*cje1516*	CDS	RNA polymerase sigma factor FliA	nonsynonymous	
G	A	*cje1554, cje1555*	upstream, downstream		.	.
G	A	*cje1562*	CDS	ATP synthase subunit alpha	nonsynonymous	c.G1237A:p.G413R
C	T	*cje1650*	CDS	amidophosphoribosyltransferase	nonsynonymous	c.G268A:p.A90T
A	G	*cje1728*	CDS	rod shape-determining protein MreB	nonsynonymous	c.A115G:p.I39V
C	T	*cje1811*	CDS	DNA-binding response regulator	nonsynonymous	c.G403A:p.E135K
C	T	*cje1821*	CDS	CmeB	nonsynonymous	c.G2003A:p.G668E
G	A	*cje1821*	CDS	CmeB	nonsynonymous	c.C1871T:p.A624V
G	A	*cje1821*	CDS	CmeB	nonsynonymous	c.C425T:p.S142F

To further confirm the role of each SNP mutant gene to the resistance phenotype, two putative resistant genes (*cje1167* and *cje1432*) were successfully inserted into the wild-type strain *C. jejuni* NCTC11168 using single gene insertion method. The *cje1167* and *cje1432* mutant genes were not successfully reintroduced into the wild type strain *C. jejuni* ATCC 33560. It was might due to the characteristics of this strain and was not easy to integrate other genes to its genome. Compared with *C. jejuni* NCTC 11168 wild type strains, a 4-fold increase in the MIC of CAP (from 2 μg/mL to 8 μg/mL) was observed for the constructed *C. jejuni* NCTC11168 + cje1432 and *C. jejuni* NCTC11168 + cje1167. The contribution of *cje1432* and *cje1167* gene in the CAP resistance of *C. jejuni* was confirmed.

### Accumulation of CAP in resistant *C. jejuni*

To evaluate the role of efflux pumps in CAP-resistant *C. jejuni*, an ultra-high performance liquid chromatography-tandem quadrupole mass spectrometry method was developed to determine the concentration of CAP in the bacteria. If efflux pumps alone could mediate the resistance, we would either not detect CAP in *C. jejuni* or the concentration of CAP would be controlled under a certain threshold regardless of how high the drug pressure was in the culture medium. In this experiment, CAP-resistant C4 strains (MIC = 256 μg/mL) were cultured on Muller-Hinton agar plates supplemented with CAP at 4, 16, or 32 μg/mL. The harvested bacteria were successively rinsed with water and methanol before extraction to ensure that the residual CAP outside the bacteria was removed. The rinsed methanol fraction was also analysed, and no CAP was detected under the analytical conditions. As shown in Fig. S1, the concentrations of CAP extracted from the bacteria were 15.4, 31.4, and 37.8 pg/mg, increasing correspondingly with the drug concentrations in the culture medium. This result indicated that CAP was not completely extruded out of cells by efflux pumps, and that there were other factors contributing to the high-level resistance.

### Alterations in the proteome of CAP-resistant mutants

**Figure 2:**
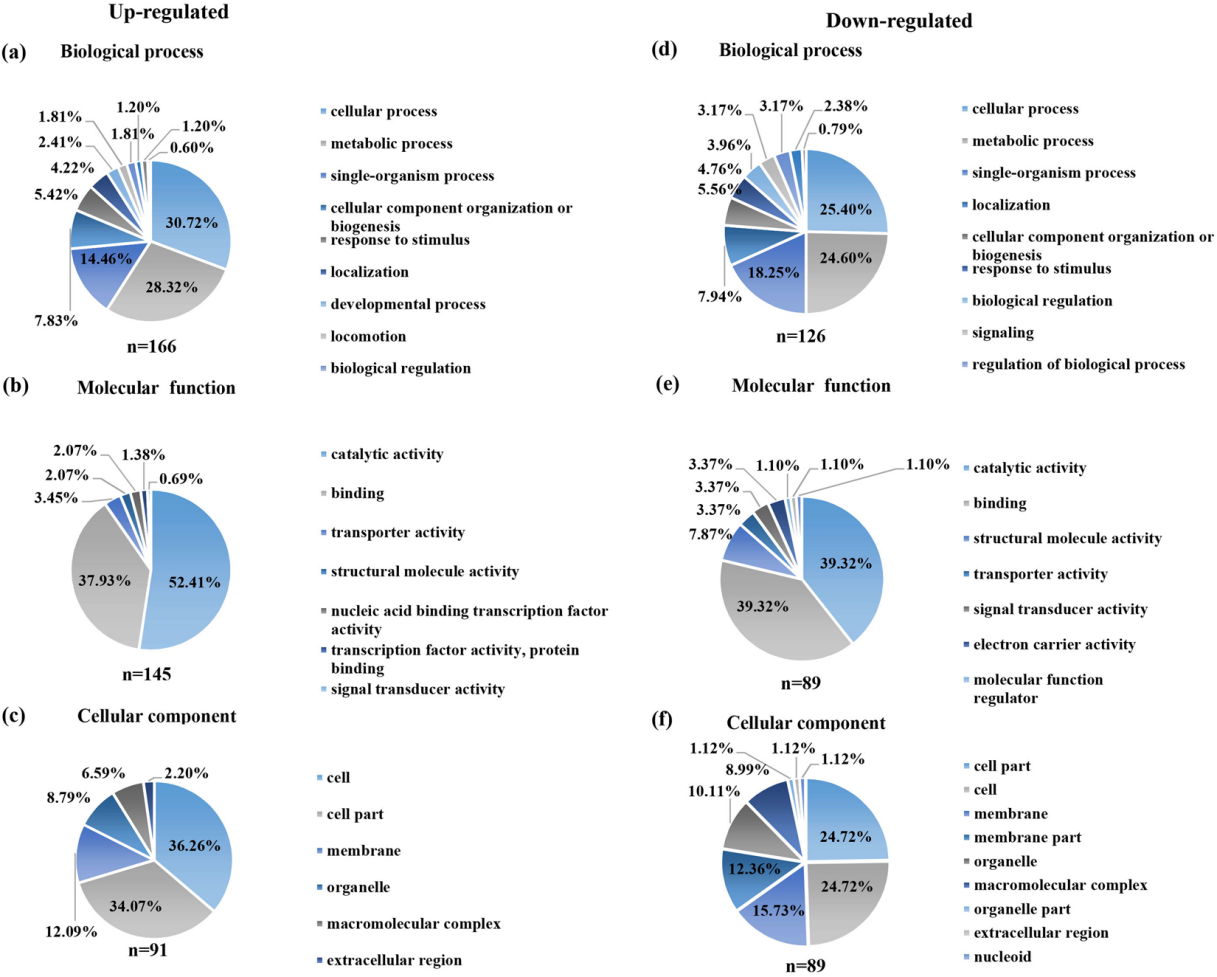
Gene ontology enrichment analysis of up-regulated and down-regulated DEPs. GO terms of up-regulated and down-regulated DEPs were categorized into biological process (**a**,**d**), molecular function (**b**,**e**), and cellular component (**c**,**f**) using Blast2GO software.

The proteomes of resistant strain and the parental strain were profiled to better understand the resistance mechanism. Using a label-free quantitative proteomics approach based on sequential window acquisition of all theoretical fragment-ion spectra mass spectrometry (SWATH-MS), we identified 1,295 proteins with a false discovery rate of <1% and quantified 1,151 proteins (Table S2). In total, 227 differentially expressed proteins (DEPs) were identified in the resistant and parental strains, including 132 up-regulated and 95 down-regulated proteins (Table S3). The DEPs were classified into three major functional ontologies (cellular component, molecular function, and biological process), using Gene Ontology (GO) enrichment analyses (Fig. 2a–f). Among the DEPs, four proteins (two up-regulated and two down-regulated) are the original products of the genes with SNPs. The concentration of the protein encoded by *cje0766* decreased dramatically, by nearly 20-fold, as expected, because a stop-gain mutation was identified in *cje0766*. Another down-regulated protein was that encoded by *cje0275*, resulting in partial recovery of antimicrobial susceptibility upon knock out. The two up-regulated proteins were cje1516 (RNA polymerase sigma factor FliA) and cje1167, which belongs to the radical SAM superfamily. To further investigate the interaction of DEPs, up-regulated (Fig. S2A) and down-regulated (Fig. S2B) DEP networks were generated using STRING 10.0 and Cytoscape 3.4.0. Using the Kyoto Encyclopedia of Genes and Genomes (KEGG) metabolic pathway analysis, the DEPs involved in major pathways were identified. The major pathways enriched by the up-regulated proteins were oxidative phosphorylation, two-component system, and ABC transporters, and for down-regulated proteins, the major pathways were ABC transporters, biosynthesis of amino acids, ribosome, and two-component system.

### Validation of SNPs and SWATH results using selected reaction monitoring assays

Considering that only four SNPs matched the DEPs, and approximately 12% of proteins of the proteome were differentially expressed, we applied a selected reaction monitoring (SRM) MS approach to confirm the protein expression of the genes with SNPs and DEPs. Fifteen DEPs from SWATH were selected for further validation in two KEGG pathways, namely, oxidative phosphorylation (six up-regulated) and ABC transporters (two up-regulated and seven down-regulated), owing to their close correlativity or possible association with drug resistance. In addition, all the original proteins of the 18 genes with substitutions in coding regions were selected for SRM validation. Next, to develop and optimise a specific SRM method for each protein, a set of peptides were selected from a collection of the shotgun proteomic analysis of *C. jejuni*. For each peptide, we tested different predicted SRM transitions and extracted up to five peptides and six transitions per peptide that resulted in the highest signals for each protein, together with peptide elution times. Finally, we successfully generated a multiplexed, time-scheduled SRM assay using 596 transitions for 33 selected proteins (Table S4).

**Figure 3:**
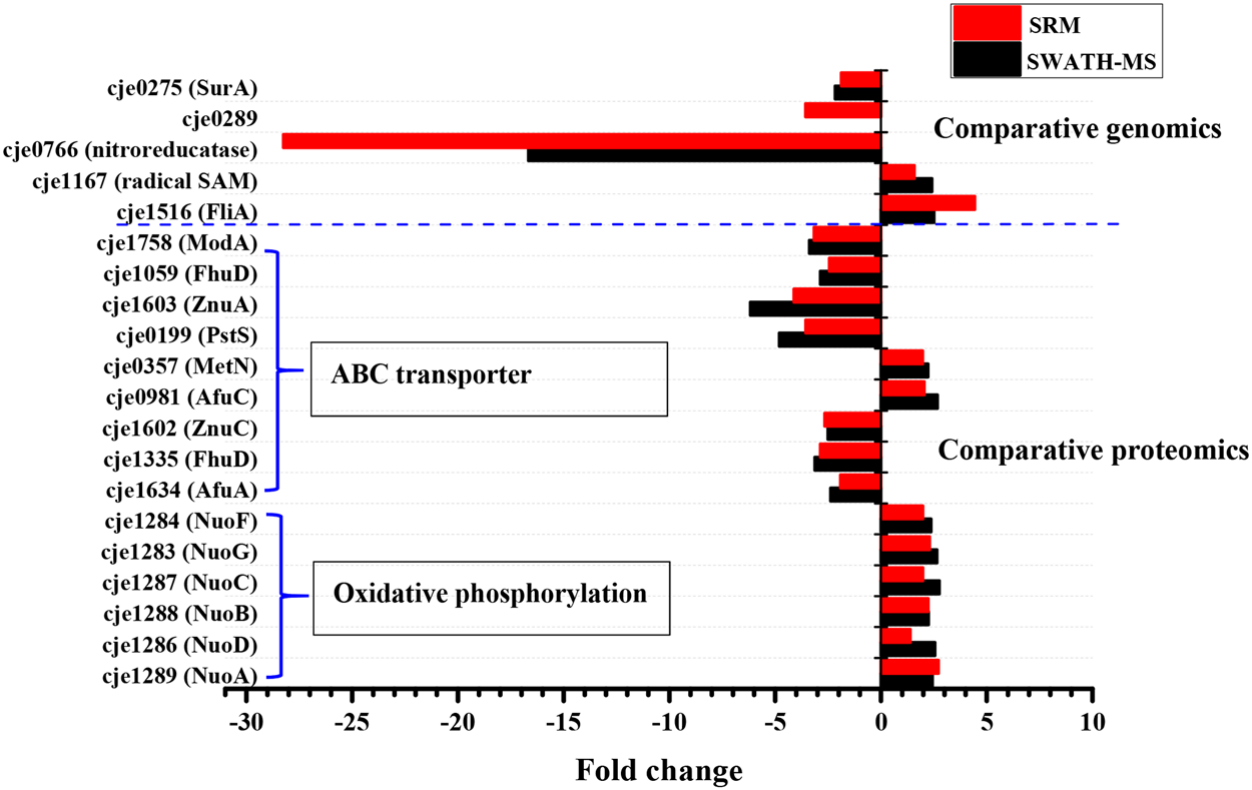
Quantification results from SWATH and SRM analyses.

We exploited the multiplexed SRM assay to measure the selected proteins from resistant strain and the parental strain. The proteins were quantified by calculating the average of multiple SRM transition intensities per peptide and multiple peptides per protein. As shown in Fig. 3, the SRM and SWATH analyses exhibited similar trends for all selected proteins, except cje0289, which could not be identified by SWATH profiling. The developed SRM method detected this protein and found that it was down-regulated in resistant strains.

### Influence of CAP resistance on bacterial metabolic pathways

**Figure 4:**
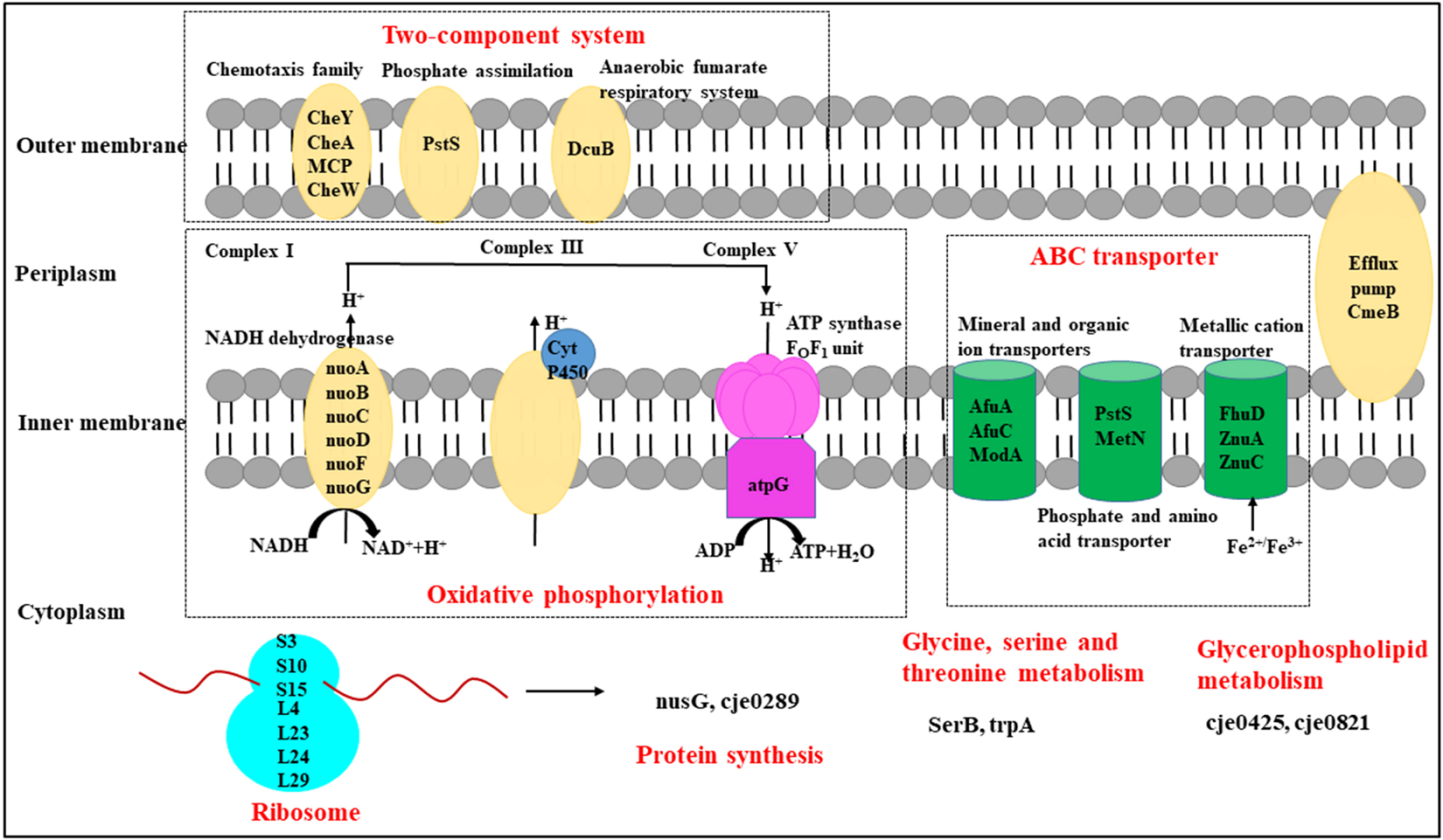
Schematic representation of disturbed metabolic pathways in CAP-resistant strains including ABC transporter, oxidative phosphorylation, ribosomal assembly, and two-component system. Disturbed metabolic pathway were indicated in RED.

SRM assays validated the SWATH profiling results and therefore confirmed specific metabolic pathways associated with resistance (Fig. 4). Both SWATH and SRM showed that cje1283, cje1284, cje1286, cje1287, cje1288, and cje1289 were up-regulated, with approximately two-fold increase, in resistant strains. These six proteins correspond to NADH-quinone oxidoreductase subunits A, B, C, D, F, and G, all of which belong to NADH dehydrogenase in the oxidative phosphorylation process. Cje0767, like cje0766, was also down-regulated by 20-fold in resistant strains, and corresponded to an NAD(P)H-dependent oxidoreductase that was not included in the KEGG pathway. These results revealed that resistant strains inactivated an unknown metabolic pathway and then relied more on NADH dehydrogenase in the oxidative phosphorylation pathway. Subsequently, we identified two additional proteins (and the corresponding genes) involved in the oxidative phosphorylation pathway that were subunits of ATP synthase. *Cje1562* (ATP synthase subunit alpha) exhibited a G1237A substitution but the protein expression was not changed, whereas the expression of cje1563 (ATP synthase subunit gamma) was increased by >two-fold. Although the specific functions and interactions of these proteins in *C. jejuni* were not very clear, collectively these findings demonstrated that the energy metabolism was adjusted in resistant strains to overcome the drug pressure.

The ABC transporters were also fully validated using SRM, including the up-regulated cje0357 (MetN) and cje0981 (FtsE) and the down-regulated cje1758 (ModA), cje1634 (AfuA), cje0199 (PstS), cje1059 (FhuD), cje1335 (FhuD), cje1603 (ZnuA), and cje1602 (ZnuC). These DEPs participated in the transport of mineral and organic ions, metallic cations, phosphates, and amino acids. In the resistant strains, the intake of molybdate, iron ion and iron complex, zinc, and phosphate was suppressed, whereas the import of d-methionine was increased.

## Discussion

Different resistance genes have been reported as conferring resistance to CAP, but few strains have been identified containing multiple CAP resistance genes. Little is known about the correlation between these various resistance genes or whether they act synergistically to confer resistance, and few studies have been conducted to investigate the responses of the whole organism to resistance, because current studies still focus only on genetic changes. In the present study, the molecular basis of CAP resistance in *C. jejuni* was characterised using integrated genomics and quantitative proteomics approaches. We discovered new resistance mutation and observed their combined contributions to bacterial resistance. In addition, we identified that the shifts of several metabolic pathways were essential to resistance in *C. jejuni*.

To unravel the role of SNPs in the resistance mechanism of *C. jejuni*, we constructed gene-knockout mutants of each gene individually, and then evaluated their phenotype of CAP resistance. We found that at least three mutant genes contributed jointly to confer high-level CAP resistance in *C. jejuni*. Three nonsynonymous substitutions were identified in the efflux transporter gene cmeB (cje1821), and knockout of this gene resulted in only partial recovery of drug susceptibility. Overexpression of these efflux transporters is typically required for mediating antibiotic resistance^20,21^, and it has recently been shown that both the sequence variation and enhanced expression of cmeABC contributed to enhancing the resistance to multiple antibiotics, including CAP, in *Campylobacte*r^22^. In this study, the SWATH proteomics data showed that cmeB expression was not changed in resistant strains, which was then validated by SRM assays, but the variant cmeB only conferred a modest level of resistance to CAP. The accumulation of CAP in resistant strains proved the existence of resistance mechanisms other than the efflux pump. Cje1432, another SNP affecting the MIC of CAP, encodes a dimethyltransferase that catalyses the transfer of methyl groups from S-adenosylmethionine (SAM) to two adjacent adenosines (A1518 and A1519) in the loop of a conserved hairpin near the 3′-end of 16 S rRNA^23^, and its mutations led to resistance against the aminoglycoside antibiotic kasugamycin in *Neisseria gonorrhoeae* ^24^. The specific function of *cje1167* has been verified in the constructed *C. jejuni* NCTC 11168 + cje1167 mutant strains using single gene insertion method. This evidence suggests that mutation of the 16 S rRNA dimethyltransferase confers resistance to CAP in *Campylobacter*. The product of the third successful knockout SNP (cje0275) is peptidyl-prolyl cis-trans isomerase, a SurA-like protein. SurA is the primary chaperone that facilitates membrane insertion of most outer membrane proteins in gram-negative bacteria^25^. Deletion of *surA* in *Escherichia coli* causes a decrease in outer membrane density and an increase in bacterial drug susceptibility, which means that the decrease of MICs in gene-knockout phenotype strains may be due to the deletion of the gene rather than the SNP. However, SWATH and SRM analyses both showed a two-fold decrease of protein expression of cje0275 in resistant strains. The mutant sequence in the protein of cje0275 may play a role in the resistance mechanism.

Cje1167 was screened out by comparative genomics, SWATH profiling, and SRM validation, showing G113A substitution in its coding sequence and up-regulated protein levels in resistant strains. Radical SAM enzymes utilise potent radical intermediates to perform an array of unusual and chemically difficult transformations, including methylation, isomerization, sulphur insertion, ring formation, anaerobic oxidation, and protein radical formation^26^. *cfr* is a well-known resistance gene belonging to the SAM superfamily^27,28^. It encodes 23 S rRNA methyltransferase and confers resistance to multiple drugs, including CAP, in bacteria. Our data suggest that, similar to *cfr*, the *cje1167* variant could also be responsible for the resistance to CAP in *Campylobacter*.

After evaluating the adjusted metabolic pathways in resistant strains, and linking them to the SNPs and related proteins, we believe that a radical SAM enzyme–mediated mechanism exists in resistant *C. jejuni*. First, we found that resistant strains promote energy accumulation for the radical enzyme reaction. Oxidative phosphorylation serves as the major route for energy metabolism in *Campylobacter*. Resistant strains disable an unknown metabolic pathway regulated by *cje0766* and *cje0767* and switch to NADH dehydrogenase complex I in the oxidative phosphorylation pathway. NADH dehydrogenase and ATP synthase were both up-regulated by two-fold. Second, the import of methionine was increased by overexpression of the ABC transporter MetN. Methionine is the immediate precursor of SAM, which is one of the major methyl-group donors in trans-methylation reactions^29^. Third, two potential radical SAM enzymes were found to be associated with the SNP genes. *Cje1432* encodes a dimethyltransferase, catalysing the methylation of 16 S rRNA through SAM, and *cje1167* belongs to a radical SAM superfamily. Given these findings, we propose that the radical SAM enzyme confers CAP resistance by methylation of rRNA, thereby providing protection from drug binding.

Western blotting used to be the gold standard for validating proteins obtained using discovery proteomics in complex samples as an orthogonal method, but it is usually restricted to the availability of antibodies when performing large-scale validation. Nowadays, SRM assays based on targeted MS have replaced western blotting for protein quantification across samples owing to its accuracy, reproducibility, and sensitivity^30^. In this study, although the fold changes varied to some degree, the change tendencies of proteins from SRM assays were consistent with those from SWATH profiling. In particular, we not only identified mutant peptides from two SNP genes (*cje0075* and *cje1811*), using SRM, which were not identified by SWATH, but also determined an SNP gene (*cje0289*) that was not detected by SWATH and even found its lower expression in resistant strains. Cje0075 is the transcription termination/antitermination protein NusG, an ubiquitous transcription elongation factor conserver in bacteria^31^, and cje1811 is a DNA-binding response regulator belonging to the OmpR family^32^. Although these three SNP proteins are involved in RNA synthesis and signal transduction, their precise role(s) in CAP resistance remains to be ascertained.

In summary, we devised a strategy in which comparative genomics and quantitative proteomics were combined to assemble a comprehensive picture of CAP resistance in *C. jejuni*. Our findings suggest that multiple genes, particularly those encoding mutant cmeB and a radical SAM enzyme, contribute jointly to confer high-level resistance to CAP. Furthermore, major metabolic pathways were identified, revealing their adjustment and impact on the resistance mechanism. However, the functions of SNPs with uncertain roles as well as those of many DEPs should be verified in future studies. Our strategy represents a significant step towards characterizing genome- and proteome-wide alterations in antibiotic-resistant bacteria, and thus opens the way to elucidate the drug resistance mechanism from a systems biology perspective.

## Methods

### Eliciting antibiotic resistance and culture conditions

To generate antibiotic-resistant mutants, the *C. jejuni* ATCC 33560 parental strain was inoculated onto multiple Muller-Hinton (MH) agar plates (Fluka, Sigma-Aldrich, St. Louis, MO), and then independent stepwise *in vitro* selection experiments were conducted for resistance to CAP. Single colonies (n = 10) were picked and tested at each stepwise selection. The bacteria were grown on MH agar plates at 42 °C under microaerobic conditions (5% O_2_, 5% CO_2_, and 90% N_2_). Antimicrobial susceptibility testing was conducted using the standard agar dilution method according to the guidelines of the Clinical and Laboratory Standards Institute^33^. *C. jejuni* ATCC 33560 was used as the quality control stain. Experiments were repeated at least three times. A high-level resistant strain C4 from a single colony was selected in the further study.

### DNA extraction, whole genome sequencing, and assembly

DNA from the CAP-resistant and parental strains was isolated using the Wizard^®^ Genomic DNA Purification Kit (Promega, Madison, WI, US). Library preparation was carried out as a 10-kb insert library using C2 chemistry and sequenced using the single molecular real-time sequencing (SMRT) technique by PacBio RS system (Pacific Biosciences, Menlo Park, CA), yielding >50× average genome coverage. *De novo* assembly of the reads was carried out using continuous long reads following the Hierarchical Genome Assembly Process workflow (PacBio DevNet; Pacific Biosciences) as available in SMRT Analysis v2.0. The assembled genome was annotated using the NR/NT, Swiss-Prot, COG, GO, and KEGG databases. The SNPs identified in resistant mutants were compared to those of the parental strain, using Mauve 2.3.1^34^ and MUMmer^35^. Putative mutations identified were filtered to remove those with a Phred-scaled quality score of <100.

### Gene-knockout and gene-knockin mutant strains construction

Knockout strains of SNP mutant loci were constructed as described previously^36^. Briefly, primers for mutant construction were designed based on the genome sequence of the resistant strain. The primers used for knockout mutant construction were shown in Table S5. The DNA fragments (upstream and downstream homologous arms) of the mutant gene were amplified using a high-fidelity DNA polymerase. The kanamycin resistance cassette (Ka) was amplified from the cloning vector pET28a. The three amplicons (upstream, downstream, and Ka) were ligated in a single microcentrifuge tube, using the Gibson Assembly Master Mix. The assembled PCR product was transformed into resistant strains by electrotransformation, and resistant colonies were selected on blood agar plates containing 50 μg/mL kanamycin.

To further confirm the role of each mutant gene to the resistance phenotype, *cje1167* and *cje1432* were selected to construct the knockin mutant strains using single gene insertion method. Briefly, the *cje1167* and *cje1432* gene were amplified from C4 mutant strains using primers pCje1167-F, pCje1432-F and pCje1167-R, pCje1432-R, respectively (Table S5 in the Supplemental Material), respectively and cloned into the pUC19 suicide vector (TaKaRa). This suicide vector was then attempted to transfer into *C. jejuni* ATCC 33560 and *C. jejuni* NCTC 11168, respectively by natural transformation. The transformants were selected on MH agar plates containing CAP (2 μg/mL). Resistant phenotype was determined to verify the function of the selected mutant genes.

### Protein lysis and digestion

Drug susceptibility tests showed that the MICs of *C. jejuni* parental strain and the constructed C4 mutant strains were 4, 256 μg/mL, respectively. The *C. jejuni* parental strain was cultured in blank MH agar, and resistant strains were cultured in MH agar supplemented with 16 μg/mL CAP. Three biological replicates were performed for each condition. Bacterial cells were harvested and washed with phosphate buffered saline twice. Then, a 0.1-g cell pellet was suspended in 1 mL of lysis buffer (7 M urea, 2 M thiourea, 4% CHAPS, 65 mM DTT, 1 mM PMSF, and 10 μL nuclease mix) for sonication for 3 min on ice. The sample was centrifuged at 30,000 × *g* for 30 min (Beckman Coulter Inc., U.S.A.), and the supernatant was subjected to proteomic analysis. Additionally, a small aliquot was taken for the Bradford Protein assay (Sangon Biotech, Shanghai). The proteins were digested based on filter-aided sample preparation protocol^37^. Briefly, bovine serum albumin was added to 200 μg of protein extract, to a final concentration of 4 pmol. Then, proteins were reduced in 8 M urea and 20 mM dithiothreitol buffer at 37 °C for 1 h and alkylated with iodoacetamide (60 mM final concentration) in the dark for 30 min. The mixture was then transferred to an ultra-filtration unit (10 kD MWCO, Sartorius) and centrifuged at 14,000 × *g* for 20 min, followed by six rounds of buffer exchange processing with 200 µL of 25 mM NH_4_HCO_3_ at 14,000 × *g* for 20 min. Trypsin (4 µg) in 100 µL of 25 mM NH_4_HCO_3_ was added to the ultrafiltration unit and incubated at 37 °C overnight. Peptides were eluted by centrifuging at 14,000 × *g* for 20 min, and then collected from the collection tube.

### Label free quantitative proteomic analysis

For shotgun analysis, samples from both the parental and resistant strains were pooled and analysed on TripleTOF^®^ 5600 + (AB SCIEX, Framingham, US), using the data independent acquisition (DIA) mode. Peptides were first loaded on a trap column, and then separated on a nano column (75 μm × 15 cm, C18, 3 μm) using an Eksigent 415 Nano LC system (AB SCIEX, Framingham, US). The flow rate was set to 300 nL/min over a 120-min multi-segment gradient on solvent B (5% dimethyl sulphoxide, 0.1% formic acid in 93% acetonitrile). Triple technical replicates were conducted with different MS full-scan ranges; for the first run, the range was set to 350–1500 m/z; whereas, for the second and third runs, it was set to 350–750 m/z and 745–1500 m/z, respectively. A top 40 MS/MS acquisition criterion was set, with an accumulation time of 75 ms for each spectrum, and the total cycle time was approximately 3.3 s. The MS data were searched using ProteinPilot 5.0 to generate the SWATH library, which contained information on targeted proteins, peptides, and their fragment ions for extraction of SWATH data.

For SWATH analysis, the generated library and data files were together loaded into the PeakView v2.2 SWATH Processing MicroApp v2.0. Proteins that met the 1% false discovery rate were selected without shared peptides. The modified peptides and those with confidence intervals <90% were excluded. Up to 20 peptides were chosen per protein. The fragment ions were automatically selected according to the following rules: (i) fragment ions of a given peptide were ranked by their intensity; (ii) ions higher in m/z than the y4 fragment ion were ranked highest; (iii) ions within the SWATH isolation window were excluded from selection; (iv) if insufficient target ions were found, ions lower than y4 but outside the SWATH window were chosen; (v) if there were still insufficient ions, then fragment ions from within the SWATH window region were chosen. Up to six transitions were chosen per peptide. Extracted ion chromatograms (XICs) were then generated for the fragment ions of each peptide and scored. The XIC extraction time window was set to 15 min, and the mass window was set to 0.05 Da. After the peptides were selected for each protein, the retention time was realigned according to 10 manually selected peptides that constantly had high signal intensities and were distributed along the entire time axis. The XIC areas of the fragment ions of a targeted peptide were summed to represent the peptide. Then, the areas of the corresponding peptides were summed to represent the targeted proteins. These areas were then used for relative quantification or further statistics analysis.

### Statistical analyses

Data normalization was conducted using MarkerView v1.3, based on the total area sums algorithm, followed by a *t*-test analysis. Relative quantitative proteins with *p*-value <0.01 and fold change >2.0 were considered as DEPs. Gene ontology enrichment analysis and functional interaction network of up-regulated and down-regulated DEPs were performed by Blast2GO and Cytoscape software.

### Targeted proteomic analyses

The SRM experiment was conducted using an Eksigent Nano LC and a QTRAP^®^ 6500 MS system (AB SCIEX, Framingham, US). The mobile phase consisted of solvent A (2% acetonitrile and 0.1% formic acid in water) and solvent B (2% water and 0.1% formic acid in acetonitrile). The trap column (200 μm × 0.5 mm) and analytical column (75 μm × 150 mm) were packed with 3-μm ChromXP C18-CL resin (Eksigent cHiPLC columns). The injection volume was 6 μL. The peptides were separated with a gradient from 5% to 20% B over 50 min, to 32% B over 20 min, and to 80% B over 5 min, at a flow rate of 0.3 μL/min. The column was then flushed with 80% B for 5 min, and re-equilibrated with 5% B for 10 min. For MS settings, the scheduled MRM type was selected with the imported MRM transition list. Other MS parameters were as follows: MRM detection window, 600 s; target scan time, 2.5 s; ion source gas 1, 15; curtain gas, 30; ionspray voltage floating, 2400. All the data were acquired using Analyst v1.6 and processed using MultiQuant™ v3.0.

### Accession number

Nucleotide sequences for *C. jejuni* ATCC 33560 genome have been deposited in the GenBank WGS database with the accession code PRJNA374478 (accession CP019838).

## Electronic supplementary material

Supplementary Material

Table S1

Table S2

Table S3

Table S4

Table S5
