# Supplementary material for: Integrated Genomic and Proteomic Analyses of High-level Chloramphenicol Resistance in Campylobacter jejuni
Source: Sci Rep. 2017 Dec 5;7:16973. doi: 10.1038/s41598-017-17321-1 (PMC5716995; doi:10.1038/s41598-017-17321-1)
Supplement: Supplementary file 1 — Supplementary Material [file 41598_2017_17321_MOESM1_ESM.doc]

**Supplementary Material**

**Integrated Genomic and Proteomic Analyses of High-level Chloramphenicol Resistance in *Campylobacter jejuni***

Hui Li^a, b^, Yingyu Wang^a^, Qin Fu^a^, Yang Wang^a^, Xiaowei Li^a^, Congming Wu^a^, Zhangqi Shen^a^, Qijing Zhang^c^, Peibin Qin^d^, Jianzhong Shen^a^,* & Xi Xia^a^,*

^a^ Beijing Advanced Innovation Center for Food Nutrition and Human Health, College of Veterinary Medicine, China Agricultural University, Beijing 100193, P. R. China.

^b^ Beijing Key Laboratory of Diagnostic and Traceability Technologies for Food Poisoning, Beijing Center for Disease Prevention and Control, Beijing 100013, P. R. China.

^c^ Department of Veterinary Microbiology and Preventive Medicine, College of Veterinary Medicine, Iowa State University, Ames, IA 50011, USA.

^d^ Shanghai AB Sciex Analytical Instrument Trading Company Limited, Beijing 100015, P. R. China.

*Corresponding author. Tel: +86-10-62732803; Fax: +86-10-62731032.

E-mail: sjz@cau.edu.cn (J. Shen), [xxia@cau.edu.cn](mailto:xxia@cau.edu.cn) (X. Xia).

**Figure S1. Drug accumulation in CAP-resistant strains cultivated in MH agar supplemented with different level of CAP.** Data presented were the mean values of three biological replicates ± SD. Asterisk denoted values that were significantly different from the corresponding cultured strain. *, *p* < 0.05.


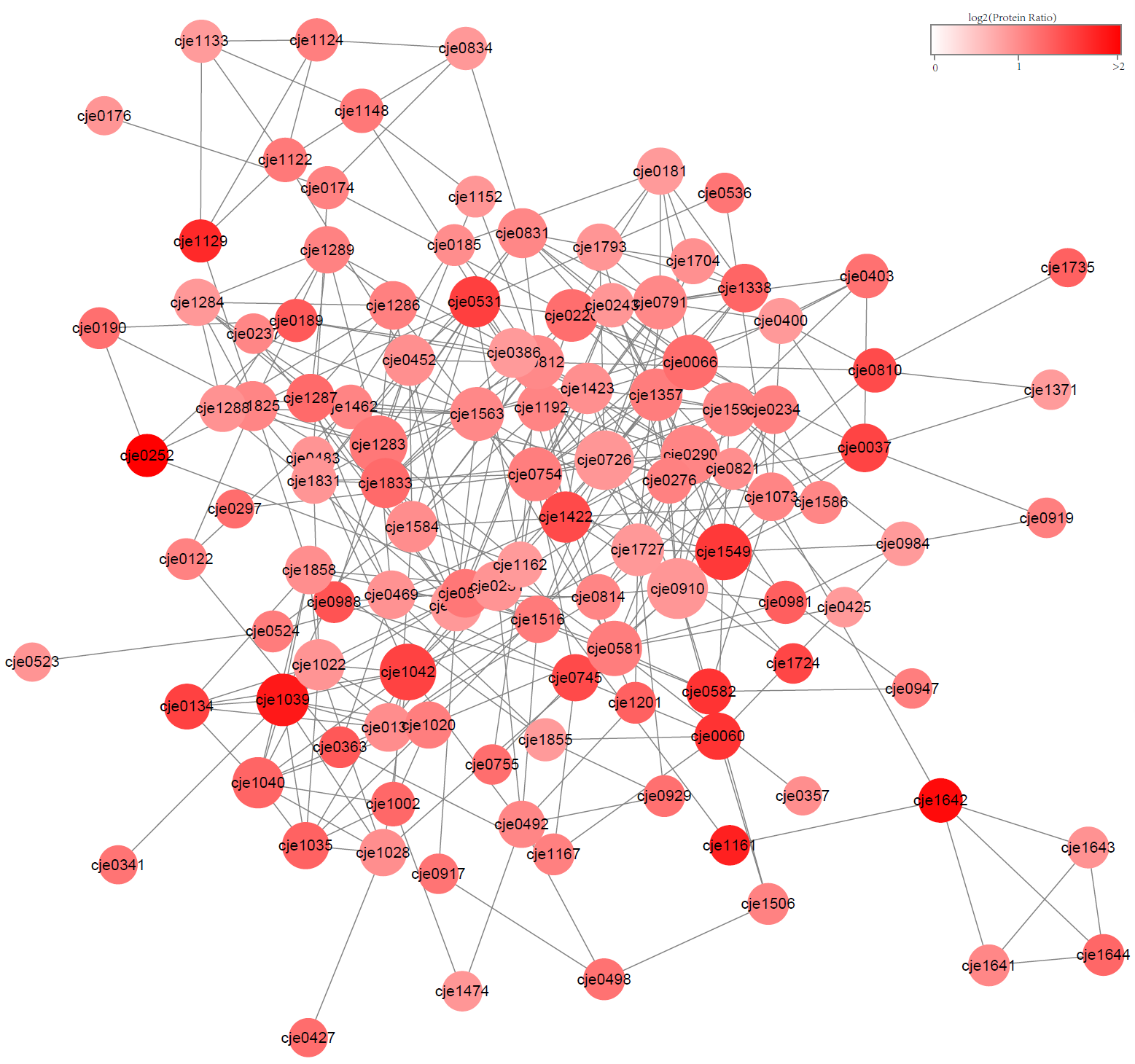

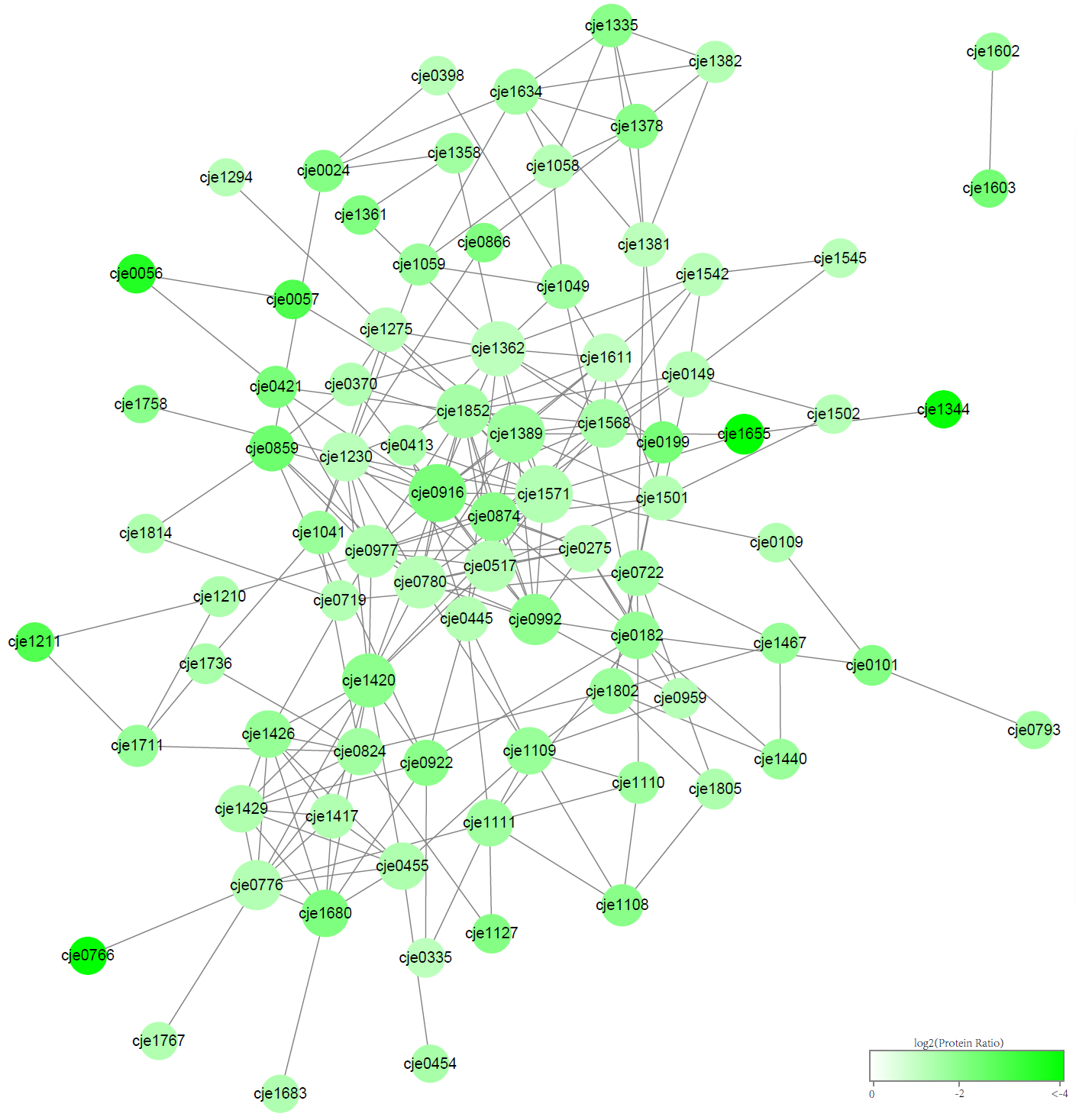


**(a)**

**(b)**

**Figure S2** Functional interaction network of up-regulated (a) and down-regulated (b) DEPs generated by STRING and Cytoscape software.
